# Widening of narrow urethral plates with lateral skin in TIP hypospadias repair: single center series

**DOI:** 10.1186/s12893-024-02400-8

**Published:** 2024-04-12

**Authors:** Yingrui Xu, Yan He, Hongwei Wang, Xuemin Wu, Zhaoquan Liu, Guoqiang Du, Xiangyu Wu, Rongde Wu, Yanze Wang, Wei Liu

**Affiliations:** 1 Department of Pediatric Surgery, Shandong Provincial Hospital, Shandong University, Jinan, Shandong China; 2 Department of Pediatric Surgery, Shandong Provincial Hospital Afiliated to Shandong First Medical University, Jinan, China

**Keywords:** Hypospadias repair, Urethral plate, Tubularized incised plate, Complications, Urinary flow rate

## Abstract

**Background:**

To compare the outcomes of hypospadias repair using tubularized incised plate (TIP) urethroplasty and modified TIP with lateral skin to widen the urethral plate (WTIP).

**Materials and methods:**

Data were obtained from pre-pubertal boys who underwent primary hypospadias repair between May 2018 and July 2023. The cases were divided into two groups; one group underwent TIP with urethral plate ≥ 6 mm width and the other group with urethral plate width < 6 mm underwent WTIP. WTIP urethroplasty was performed by widening incisions on the outer margins of the urethral plate to incorporate penile and glandular skin lateral to the urethral plate to facilitate tubularization. Complication rates and urinary functions were compared.

**Results:**

A total of 157 patients were enrolled in this study. Eighty-eight cases with narrow urethral plate were subjected to WTIP urethroplasty, and the rest were subjected to TIP urethroplasty. The preoperative glans width in WTIP group was less than that in TIP group (*P* < 0.001), and 44.3% had midshaft meatus in WTIP group compared to 17.4% in TIP group (*P* < 0.001). However, the incidences of postoperative complications (17.6% vs. 21.6%, *P* = 0.550) were not statistically different between the TIP and WTIP groups. In addition, both groups did not differ significantly in postoperative uroflowmetry assessment.

**Conclusions:**

The described technique helps to create an adequately caliber aesthetic neomeatus and facilitates tubularization, especially in hypospadias with a narrow urethral plate. Our data suggest that augmentation of a narrow urethral plate with WTIP has a similar surgical outcome to that of the TIP procedure in patients with a wide urethral plate.

## Introduction

Hypospadias is one of the most common congenital disorders of the urogenital system, with an incidence of approximately in 1/300 [1]. Surgery is the only effective treatment for patients with hypospadias. Since its introduction by Snodgrass in 1994, tubularized incised plate (TIP) urethroplasty has rapidly become one of the most widely used techniques for hypospadias repair [2]. However, a urethral plate of sufficient width and quality is essential for TIP procedures [3] as the width of the urethral plate was believed to be associated with complications after TIP repair [4, 5]. Several studies of urethral plate indicated that the narrow urethral plate was related to postoperative complications, including fistula (12.5 − 54.6%), meatal stenosis (4.2 − 17.5%), and glans dehiscence (4.5 − 20.0%) [4, 6–8]. Hence, modified TIP procedures were introduced for patients with narrow urethral plates [8, 9]. Studies on these techniques have shown that widening the urethral plate is safe and helpful in reducing the incidence of postoperative complications. In this study, we proposed a widened urethral plate-modified TIP (WTIP) technique using skin from both sides in patients with hypospadias with narrow urethral plate, and determined the surgical and functional outcomes.

## Methods and materials

We retrospectively analyzed the clinical data of patients with hypospadias who underwent TIP or WTIP repair at our institution between May 2018 and July 2023. The exclusion criteria were the use of androgen stimulation and a combination of disorders of sexual development (DSD). None of the children had undergone any penile surgery, including circumcision. TIP was performed when the urethral plate width was ≥ 6 mm and WTIP was used if the urethral plate width was < 6 mm. Clinical data, including age at surgery, meatal location, preoperative glans width, preoperative urethral plate width, mean width of the urethral plate after midline incision, and length of urethroplasty were recorded.

### Description of surgical technique

**Fig. 1:**
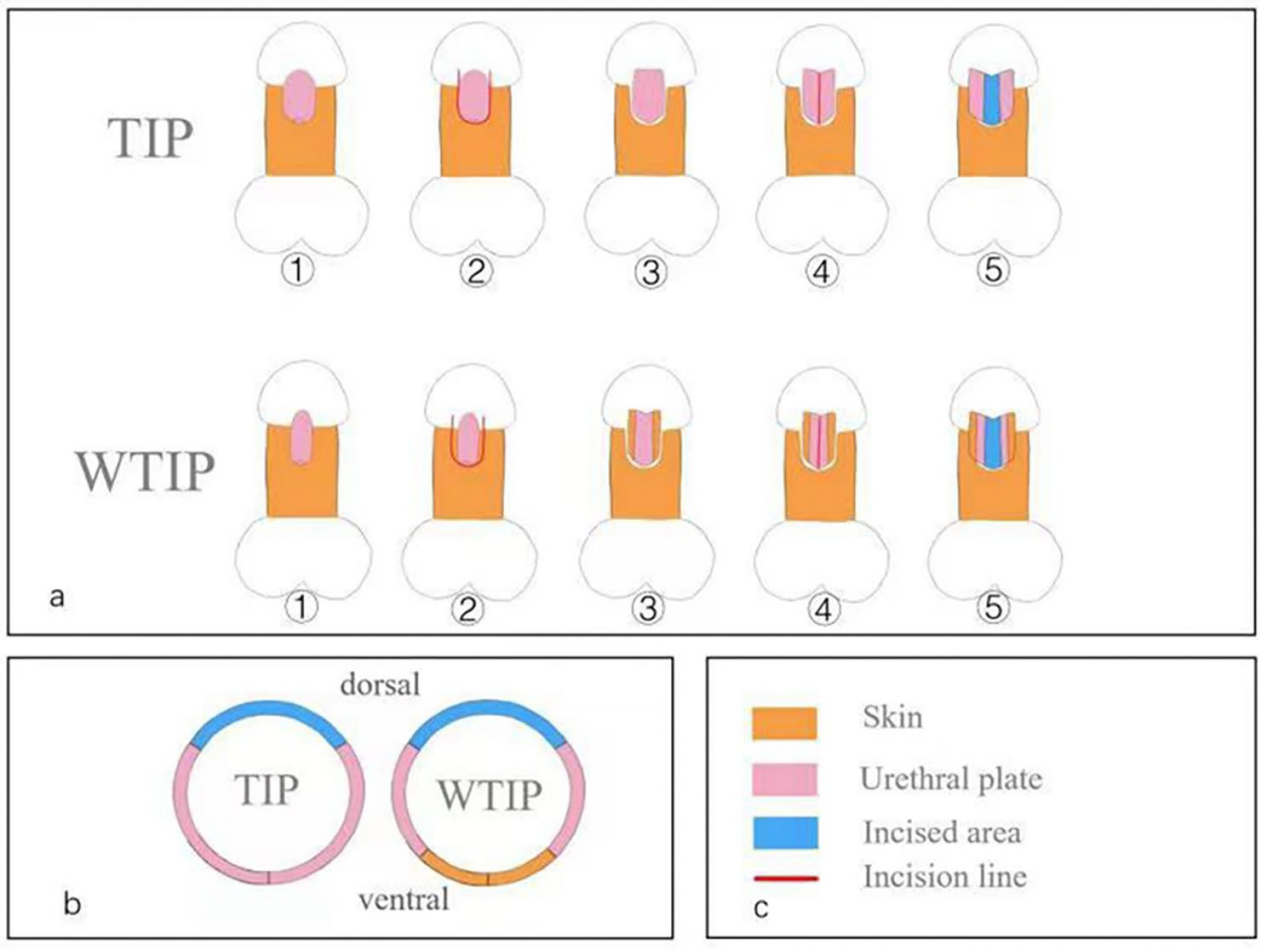
Schematic diagram of traditional TIP and WTIP. (**a**) Different procedures for widening urethral plate in two groups. (**b**) Cross section view after urethroplasty. (**c**) Color annotation

The TIP procedure was consistent with what snodgrass described in the literature [10]. For the WTIP, a U-shaped incision was performed some milimeters on the skin away from the both sides of the urethral plate mucosa to obtain an adequate urethral plate (Fig. 1). And then, the WTIP procedure is similar to the TIP. All patients underwent degloving to correct penile curvature. A midline incision of the urethral plate was made, a suitably sized silicone urethral catheter was inserted, and the urethra was coiled. In all the patients, the urethra was sutured in two layers with 7 − 0 Coated Vicryl Plus Antibacterial Absorbable Suture, the first using interrupted sutures and the second a running suture. A dartos pedicle flap is dissected from the preputial hood and dorsal shaft skin, then transposed ventrally to cover the entire urethra. Finally, the skin of the glans and the penis were sutured.

### Postoperative treatment and follow-up

In all patients, the hydrocolloid dressing, and urinary catheter were removed 6–7 days and 3–4 weeks after operation respectively. Outpatient follow-up was performed at 1, 3, 6, and 12 months postoperatively, and once a year thereafter. The main contents of postoperative follow-up included observing the recovery of the appearance of the penis, recording the occurrence of postoperative complications (including urethral fistula, meatal stenosis, and glans dehiscence) and measuring the postoperative urine flow rate (at least 6 months after surgery).

### Statistical analysis

SPSS version 25 for Windows was used for the statistical analysis. Numerical variables are described as median and interquartile range (IQR) and analyzed using the rank sum test. The chi-square test was used to analyze categorical variables and statistical significance was set at *P* < 0.05.

## Results

A total of 157 patients with a median (IQR) age of 37.03 (26.57, 53.69) months were enrolled in this study. The TIP and WTIP procedures were performed in 69 and 88 patients, respectively. There were no statistically significant differences in age at surgery, mean width of the urethral plate after midline incision, or length of urethroplasty between the two groups. In WTIP group, the proportion of midshaft meatus was significant more than that in the TIP (44.3% vs. 17.4%, χ^2^ = 12.805, *P* = 0.002). The preoperative glans width in WTIP group was significant smaller than that in TIP group, and the median (inter-quartile range) of the preoperative glans width was 14.00 (13.00,15.00) vs. 15.00 (14.00,16.00) for WTIP and TIP (Z=-4.408, *P*<0.001) (Table 1).

**Table 1:** Characteristics of patients in studied groups

Characteristics	TIP (*n* = 69)	WTIP (*n* = 88)	Z/χ^2^/t	*P*
Age at operation, M (P_25_, P_75_), months	38.70 (28.97, 68.74)	34.94 (25.88, 46.13)	-1.652	0.099
Meatus location^a^, N (%)				
Distal	57 (82.61%)	49 (55.68%)	12.786	< 0.001
Midshaft	12 (17.39%)	39 (44.32%)		
Preoperative glans width, M (P_25_, P_75_), mm	15.00 (14.00,16.00)	14.00 (13.00,15.00)	-4.408	< 0.001
Preoperative urethral plate width, M (P_25_, P_75_), mm	7.00 (6.00, 7.15)	4.67 (3.71, 5.23)	-9.106	< 0.001
Mean width of urethral plate after median incision, M (P_25_, P_75_), mm	12.00 (11.00,13.00)	12.00 (11.00,12.88)	-1.023	0.306
Length of urethroplasty, $\stackrel{-}{x} \pm s$, mm	15.77 ± 4.17	15.98 ± 5.32	-0.276	0.783

^a^ Aaccording to Barcat classification

The median (IQR) follow-up time for the 157 patients was 7.13 (2.82–15.17) months. The incidences of postoperative complications (17.6% vs. 21.6%, *P* = 0.550), urethral fistula (14.5% vs. 14.8%, *P* = 0.961), meatal stenosis (1.4% vs. 3.4%, *P* = 0.631), and glans dehiscence (2.9% vs. 4.5%, *P* = 0.696) were not statistically different between the TIP and WTIP groups (Table 2).

**Table 2:** Comparison of complications between the two groups, N (%)

Complications	TIP (*n* = 69)	WTIP (*n* = 88)	χ^2^	*P*
Urethral fistula	10 (14.49%)	13(14.77%)	0.002	0.961
Meatal stenosis	1 (1.45%)	3 (3.41%)	0.598	0.631^a^
Glans dehiscence	2 (2.90%)	4(4.55%)	0.285	0.696
Total	12 (17.39%)	19 (21.59%)	0.430	0.550

^a^ Fisher’s exact test

A total of 65 patients completed the uroflowmetry assessment postoperatively and the results showed no statistical difference in maximum flow rate (Qmax) and average flow rate (Qave) between the two groups (*P* > 0.05, Table 3).

**Table 3:** Analysis of postoperative flow rate in both groups, $\stackrel{-}{x} \pm s$

Variable	TIP (*n* = 28)	WTIP (*n* = 37)	*t*	*P*
Maximum flow rate (ml/s)	6.99 ± 2.68	6.92 ± 2.37	0.101	0.920
Average flow rate (ml/s)	4.63 ± 1.52	4.53 ± 1.55	0.240	0.811

## Discussion

The greatest advantage of the TIP procedure is that the tube is coiled using its own urethral plate and no additional urethral graft is required [11]. However, there are still significant complications associated with this procedure, including urethral fistula, meatal stenosis, and glans dehiscence [2, 12]. It has been reported that urethral plate width < 8 mm was associated with higher postoperative urethral fistula and meatal stenosis [4, 13]. Ali MM et al. analysed 40 patients with distal primary hypospadias with narrow urethral plate, and the result showed that the incidence of complications was 37.5% including glandular dehiscence (20%), fistulas (12.5%), and narrow meatus (17.5%) [6]. In the study of Güler Y, the rate of urethrocutaneous fistula was statistically significantly higher in patients with unfavourable urethral plate (narrow) compared to patients with wide plate (30.9% vs. 4.7%) [7]. Ru et al. showed that the average preoperative urethral plate width in Chinese children was 5.3 mm [14], and Zhang et al. found that TIP was associated with higher urethroplasty complications when the urethral plate width was < 6 mm [11]. Therefore, further improvements in surgical approaches for patients with narrow urethral plates are required to achieve satisfactory results.

Dorsal inlay graft urethroplasty is widely used to repair hypospadias with narrow urethral plates [15–17]. There are several modified TIP procedures for treating hypospadias when the width of the urethral plate is insufficient. Patankar et al. proposed a “wide skeletonization” TIP procedure, in which the distal urethral plate was “V”-shaped instead of “U”-shaped. They concluded that this procedure further helped to reduce the incidence of urethral strictures without increasing the rate of other postoperative complications [8]. Elbaky et al. compared the TIP procedure and tubularization of intact and laterally augmented plates. Tubularization was superior to the TIP procedure in terms of urethral fistula, urethral stricture, and rate of successful surgeries [9]. In this study, we designed a procedure using the lateral skin to obtain a sufficiently wide urethral plate. This procedure allows greater incisional tension, more complete healing of the coiled tube, and reduced susceptibility to urethral fistulas. After making a midline incision along the plate, it was easier to roll and suture the tube with the urethral plate and skin for urethroplasty. Our procedure ensured that the majority of urethral components after urethroplasty were derived from the original urethral plate, because healing after TIP occurs with creeping of the surface epithelium. Moreover, this technique is not limited to distal hypospadias but is also suitable for middle hypospadias while being easy to perform as no additional grafts were needed.

Previous studies showed that there were significantly higher postoperative complications in children with narrow urethral plates [4, 11, 13]. Increased collagen deposition, which increases postoperative complications, has been reported in children with urethral plate widths < 6 mm [8]. Another mechanism by which complications occur after the TIP procedure is the tendency for contraction and shrinkage of the two raw area surfaces after the dorsal midline incision in the process of healing [18–20]. As a result, the width of the dorsal incision decreases after complete epithelialization, which is why a narrow urethral plate is associated with urethral stenosis after the TIP procedure [21, 22]. Similarly, these kinds of contraction and shrinkage increased the tension on the ventral suture line during primary healing, and postoperative urethral fistulas are more likely to occur. In our study, the WTIP procedure used the skin around the urethral plate in combination with the urethral plate for coiling, meaning that the dorsal incision was shallower. Thus, collagen deposition and the tendency for contraction and shrinkage of the surfaces of the two raw areas after dorsal midline incision were reduced. Our data also suggest that the WTIP procedure in patients with a narrow urethral plate can achieve good results, in terms of postoperative complications or urinary flow rate, similar to TIP repair in patients with a wide urethral plate.

The limitations of our study include the small sample size and relatively short postoperative follow-up period. A larger cohort with a longer follow-up period is necessary to comprehensively evaluate the WTIP.

## Conclusion

The described WTIP urethroplasty helped in creating an adequate caliber aesthetic neomeatus and facilitated tubularization, especially in hypospadias patient with a narrow urethral plate, which led to a functional outcome similar to that of the TIP procedure in patients with a wide urethral plate.

## Data Availability

The data that support the findings of this study are not openly available due to reasons of sensitivity and are available from the corresponding author upon reasonable request.

## Abbreviations

TIP: Tubularized incised plate
WTIP: A widened urethral plate-modified TIP
